# Trends in Mortality from Stroke in Latin America and the Caribbean, 1979–2015

**DOI:** 10.5334/gh.1114

**Published:** 2022-04-07

**Authors:** Álvaro Soto, Francisco Guillén-Grima, Gladys Morales, Sergio Muñoz, Inés Aguinaga-Ontoso, Jairo Vanegas

**Affiliations:** 1 Departamento de Especialidades Médicas, Facultad de Medicina, Universidad de La Frontera, Temuco, CL; 2 Unidad de Neurología. Hospital Dr. Hernán Henríquez Aravena, Temuco, CL; 3 Centro de Excelencia en Capacitación, Investigación y Gestión para la Salud Basada en Evidencia (CIGES), Facultad de Medicina, Universidad de La Frontera, Temuco, CL; 4 Centro de Investigación en Epidemiología Cardiovascular y Nutricional (EPICYN), Facultad de Medicina, Universidad de La Frontera, Temuco, CL; 5 Departamento de Ciencias de la Salud, Universidad Pública de Navarra, Pamplona, Navarra, ES; 6 Instituto de Investigación Sanitaria de Navarra (IDISNA), Pamplona, Navarra, ES; 7 Medicina Preventiva, Clínica Universidad de Navarra, Pamplona, Navarra, ES; 8 Departamento de Salud Pública, Facultad de Medicina, Universidad de La Frontera, Temuco, CL; 9 Facultad de Ciencias Médicas, Departamento de Salud Pública. Universidad de Santiago de Chile, CL

**Keywords:** stroke, Latin America, Caribbean, joinpoint regression analysis, mortality, trends

## Abstract

**Background:**

Stroke is the second largest single cause of death and disability in Latin America and the Caribbean (LAC). There have been large overall declines in stroke mortality rates in most LAC countries in recent decades.

**Objective:**

To analyze trends in mortality caused by stroke in LAC countries in the period 1979–2015.

**Methods:**

We extracted data for age-standardized stroke mortality rates per 100,000 in LAC for the period 1979–2015 from the World Health Organization Mortality Database. Joinpoint regression was used to analyze the trends and compute the annual percent change (APC) in LAC as a whole and by country. Analyses were conducted by gender, region and World Bank income classification.

**Results:**

Mortality from stroke has decreased in LAC over the study period by an average APC of –1.9%. Most countries showed significant downward trends, with the sharpest decreases in Chile, Colombia and Uruguay. We recorded statistically significant decreases of –1.4% and –2.4% in mortality rates in men and women, respectively, in the whole LAC. Southern and high-income countries showed the steepest decreases.

**Conclusions:**

Stroke mortality has decreased in LAC, in both sexes, especially in southern and high-income countries. Our results could serve as a reference for the development of primary prevention and acute management of stroke policies focused on countries with higher mortality.

## Introduction

Stroke is the second leading cause of death and disability in Latin American and Caribbean (LAC), with variable incidence and prevalence throughout the continent reflecting regional socioeconomic differences [1,2]. There are also differences in the prevalence of the main cerebrovascular risk factors such as hypertension, age structure and diabetes among LAC countries [3].

LAC was a region known for a relatively low proportion of ischemic stroke (57%) compared to high-income countries (80–85%), but with a high proportion of intracerebral hemorrhage (27%) and subarachnoid hemorrhage (15%) [4]. However, recent population-based studies by Cabral et al. in Brazil and Lavados et al. in Chile have shown an increased proportion of ischemic stroke and a lower frequency of intracerebral hemorrhage in the last few decades [5,6].

Stroke incidence varies from 90 to 120/100,000 people in Central Latin America to 121 to 150/100,000 people in Andean Latin America [1]. Despite similarities in language and socioeconomic characteristics, the region differs in cultural, genetic and ethnic characteristics [7,8]. On the other hand, according to the INTERSTROKE Study, South America has a higher prevalence of hypertension (63.4%), waist-to-hip ratio (40.8%) and apoliproteins B/A1 (45.3%) as well as a lower prevalence of regular physical activity (12.9%) in comparison with North America, Europe and Australasia [9].

Stroke mortality decreased between 1970 and 2017 [10,11]; however, the absolute number of people with incident strokes has significantly increased by 81% from 1990 to 2017, the number of people who survived by 95%, and the number of those who died from stroke by 40% [11].

There are considerable differences in stroke mortality among LAC countries. In 2017, the highest age-standardized mortality rates were reported in Paraguay (67 cases per 100,000 population), whereas the lowest levels were reported in Colombia and Peru (25–29 cases per 100,000 population) [11]. In Latin American countries, from 1990 to 2017, stroke age-adjusted mortality decreased from 89.7 to 47.2 (–47% change). However, the absolute number of people who suffered a stroke increased from 184,400 to 258,900 (+40% change) [11].

The aim of this study was to analyze trends in mortality caused by stroke in LAC in the period 1979–2015.

## Methods

Age-standardized death rate (ASDR) data for men and women, without age limits, from LAC countries in the period 1979–2015 were extracted from the WHO Mortality Database (*https://apps.who.int/healthinfo/statistics/mortality/whodpms/*) [12]. The database was updated in May 2018. Due to the study design, no approval by an institutional review board was needed.

LAC countries and their available data included: Antigua and Barbuda (1985–2015), Argentina (1979–2015), Bahamas (1980–2013), Barbados (1979–2013), Belize (1980–2015), Brazil (1979–2015), Chile (1980–2015), Colombia (1984–2015), Costa Rica (1980–2014), Cuba (1979–2015), Ecuador (1979–2015), El Salvador (1981–2014), Grenada (1985–2015), Guatemala (1979–2015), Guyana (1979–2013), Jamaica (1980–1983, 2000–2011), Mexico (1979–2015), Nicaragua (2010–2015), Panama (1979–2015), Paraguay (1979–2014), Saint Lucia (1979–2014), St. Vincent and the Grenadines (1982–2015), Suriname (1979–2014), Trinidad and Tobago (1979–2011), Uruguay (1980–2015) and Venezuela (1979–2013).

The database did not have mortality rates from Bolivia, Dominica, Dominican Republic, Haiti, Honduras, Peru and St. Kitts and Nevis. Unfortunately, data for one or more calendar years were missing from a few countries. No extrapolation was made for missing data. We assumed that trends would not vary notably in those countries with few missing data. Conversely, in those countries with more missing data (Jamaica, Nicaragua), results could have been affected to a certain extent and should be interpreted carefully.

We also conducted an analysis to assess regional mortality trends, distinguishing three LAC regions: Caribbean (Antigua and Barbuda, Bahamas, Barbados, Cuba, Grenada, Jamaica, St Lucia, St Vincent, Trinidad and Tobago); Central America (Belize, Costa Rica, El Salvador, Guatemala, Mexico, Nicaragua and Panama); and South America (Argentina, Brazil, Chile, Colombia, Ecuador, Guyana, Paraguay, Suriname, Uruguay and Venezuela). According to the World Bank income classification, LAC countries were classified in two regions: high-income (Antigua and Barbuda, Bahamas, Barbados, Chile, Panama, Trinidad and Tobago and Uruguay), and middle-income (Argentina, Belize, Brazil, Colombia, Costa Rica, Cuba, Ecuador, El Salvador, Grenada, Guatemala, Guyana, Jamaica, Mexico, Nicaragua, Paraguay, Saint Lucia, St Vincent, Suriname and Venezuela).

We used the Joinpoint regression software (version 4.8.0.1; Surveillance Research Program, USA National Cancer Institute, Bethesda, MD, USA) to analyze significant changes in mortality trends. This analysis identified inflection points (called ‘joinpoints’), where there was a significant change in the linear slope of the trend. The number and location of significant joinpoints for each country and region were determined by the software using a log-linear model [13].

We computed the estimated annual percent change (APC) and corresponding 95% confidence intervals to describe the magnitude of change for each of the trends identified. In this model, age-standardized mortality rates were used as the dependent variable and year of death as the independent variable, with an annual interval type and assuming a constant variance (homoscedasticity). In all analyses, P < 0.05 was regarded as statistically significant. We also calculated the average annual percent change (AAPC) for the overall period (1979–2015) in LAC as a whole and by country. Analyses were conducted by gender and by LAC region. The Joinpoint regression analysis has been used extensively in previous research on trends in cerebrovascular diseases [10,14,15].

## Results

Between 1979 and 2015, the number of recorded deaths from stroke in 26 LAC countries increased steadily from 110,873 (56,024 men and 54,849 women) to 203,653 (99,252 men and 104,401 women).

**Figure 1:**
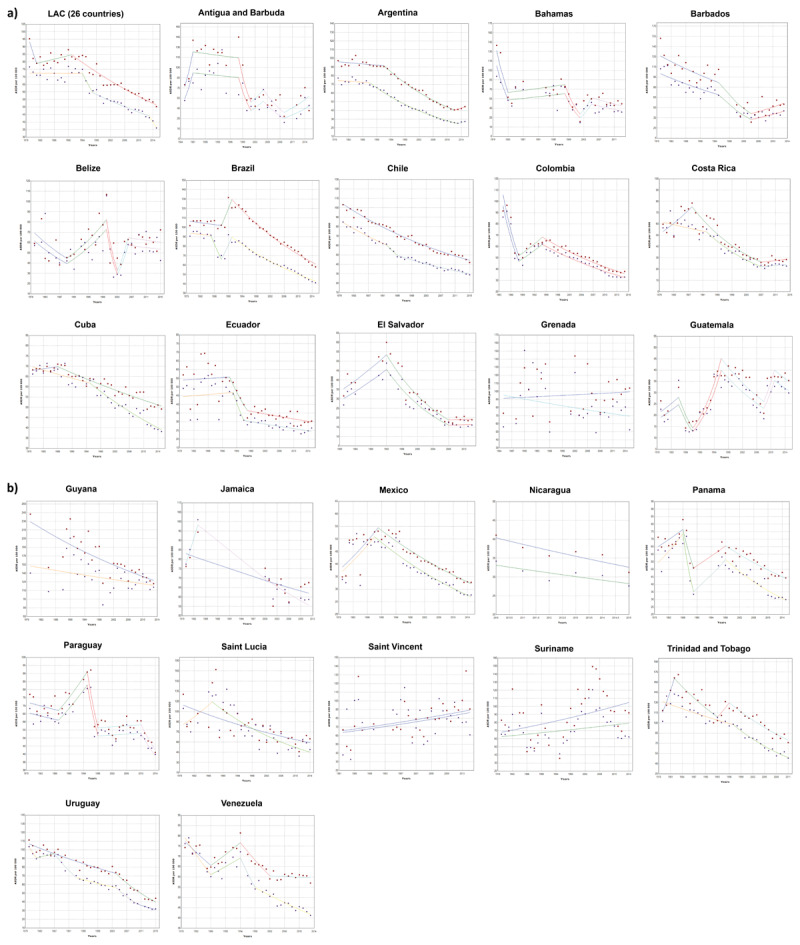
Trends in mortality from stroke in America and the Caribbean, 1979–2015. 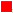, men (age-standardized mortality rated); 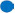, women (age-standardized mortality rated).

In 1979, the highest stroke mortality rate was found in Guyana (165.2 per 100,000) and the lowest in Guatemala (24.5 per 100,000). In 2015, Grenada had the highest mortality rate (75.3 per 100,000), whereas the lowest rate was observed in Ecuador (28.4 per 100,000) (***Figure 1***). The overall ASDR decreased from 75.1 to 36.8 per 100,000.

During the period 1979–2015 the entire LAC region showed a statistically significant decrease of –1.9% in the AAPC in mortality rates, with two joinpoints, 1992 and 2010 (***Table 1***). Mortality rates decreased significantly in 16 LAC countries. Chile, Colombia, and Uruguay had the largest decreases (AAPC, –2.8%, –3.4% and –3.0%, respectively). Only St Vincent showed a significant increase of 0.9%.

**Table 1:** Joinpoint analysis for stroke mortality trends in Latin America and the Caribbean, 1979–2015.


	TOTAL STUDY PERIOD		PERIOD 1		PERIOD 2		PERIOD 3	
			
	YEARS	APC (95% CI)	YEARS	APC (95% CI)	YEARS	APC (95% CI)	YEARS	APC (95% CI)

LAC	1979–2015	–1.9* (–2.4, –1.5)	1979–1992	–0.5 (–1.1, 0.1)	1992–2010	–2.3* (–2.7, –1.9)	2010–2015	–4.3* (–6.8, –1.7)

Antigua and Barbuda	1985–2015	–1.5 (–5.5, 2.8)	1985–1992	6.6 (–1.7, 15.6)	1992–2009	–7.3* (–9.6, –5.0)	2009–2015	7.0 (–11.7, 29.6)

Argentina	1979–2015	–2.5* (–3.0, –2.1)	1979–1990	–0.5 (–1.1, 0.1)	1990–2012	–4.1* (–4.3, –3.9)	2012–2015	2.0 (–2.8, 7.0)

Bahamas	1980–2013	–2.2* (–3.3, –1.1)						

Barbados	1979–2013	–2.3 (–5.0, 0.4)	1979–1995	–2.4* (–3.7, –1.2)	1995–2004	–7.7 (–16.9, 2.6)	2004–2013	3.5* (0.4, 6.7)

Belize	1980–2015	0.8 (–3.7, 5.6)	1980–2000	1.3 (–0.4, 2.9)	2000–2003	–12.7 (–49.2, 49.9)	2003–2015	3.8* (0.6, 7.2)

Brazil	1979–2015	–2.0* (–2.8, –1.1)	1979–1988	–2.1* (–3.0, –1.1)	1988–1991	7.0 (–3.4, 18.6)	1991–2015	–3.0* (–3.2, –2.8)

Chile	1980–2015	–2.8* (–3.5, –2.1)	1980–2008	–2.9* (–3.1, –2.7)	2008–2012	–0.1 (–5.1, 5.2)	2012–2015	–6.0* (–10.7, –1.0)

Colombia	1984–2015	–3.4* (–4.5, –2.3)	1984–1988	–16.3* (–21.4, –10.9)	1988–1994	4.7* (0.2, 9.4)	1994–2015	–3.0* (–3.5, –2.5)

Costa Rica	1980–2014	–2.2* (–3.2, –1.1)	1980–1988	2.2 (–0.8, 5.2)	1988–2008	–5.1* (–5.8, –4.3)	2008–2014	2.0 (–2.5, 6.8)

Cuba	1979–2015	–1.2* (–1.4, –0.9)	1979–1988	–0.0 (–1.0, 1.0)	1988–2015	–1.5* (–1.7, –1.4)		

Ecuador	1979–2015	–1.7 (–5.0, 1.8)	1979–1993	0.2 (–1.7, 2.2)	1993–1996	–12.5 (–42.3, 32.7)	1996–2015	–1.2 (–2.4, 0.0)

El Salvador	1981–2014	–1.9* (–3.0, –0.7)	1981–1992	3.7* (1.6, 5.9)	1992–2008	–6.3* (–7.4, –5.1)	2008–2014	0.3 (–4.3, 5.0)

Grenada	1985–2015	–0.4 (–1.5, 0.7)						

Guatemala	1979–2015	0.6 (–1.9, 3.2)	1979–1988	–5.0 (–10.3, 0.7)	1988–1995	13.1* (2.1, 25.2)	1995–2015	–0.9 (–2.5, 0.8)

Guyana	1979–2013	–1.1* (–1.8, –0.5)						

Jamaica	1980–2011	–1.1* (–1.7, –0.6)						

Mexico	1979–2015	–0.7* (–1.4, –0.1)	1979–1989	3.2* (1.1, 5.2)	1989–2015	–2.2* (–2.7, –1.7)		

Nicaragua	2010–2015	–3.7* (–6.5, –0.7)						

Panama	1979–2015	–1.5 (–3.3, 0.3)	1979–2005	–0.8* (–1.5, –0.1)	2005–2011	–5.2 (–12.5, 2.8)	2011–2015	–0.7 (–11.4, 11.3)

Paraguay	1979–2014	–0.8 (–2.7, 1.3)	1979–1995	1.4* (0.4, 2.5)	1995–1998	–10.2 (–29.1, 13.8)	1998–2014	–1.1* (–2.0, –0.2)

Saint Lucia	1979–2014	–2.2* (–2.9, –1.5)						

St. Vincent	1982–2015	0.9* (0.0, 1.8)						

Suriname	1979–2014	–0.6 (–2.6, 1.5)	1979–1995	–2.5* (–4.9, –0.1)	1995–2004	10.4* (4.4, 16.8)	2004–2014	–6.7* (–10.3, –2.9)

Trinidad and Tobago	1979–2011	–2.0* (–3.0, –0.9)	1979–1981	12.8 (–3.8, 32.3)	1981–1999	–2.0* (–2.6, –1.5)	1999–2011	–4.2* (–5.1, –3.3)

Uruguay	1980–2015	–3.0* (–3.8, –2.2)	1980–2005	–2.2* (–2.4, –1.9)	2005–2012	–6.5* (–9.2, –3.7)	2012–2015	–1.5 (–7.8, 5.3)

Venezuela	1979–2013	–1.5* (–2.3, –0.6)	1979–1986	–3.0* (–5.0, –1.0)	1986–1992	1.6 (–2.8, 6.2)	1992–2013	–1.8* (–2.2, –1.4)


APC, annual percent change; CI, confidence interval. * P < 0.05 for change in trend.

### Trends of mortality from stroke by gender

Stroke mortality trends for men are presented in ***Table 2***. In 1979, the highest rates were observed in Guyana (242.3 per 100,000), and the lowest in Guatemala (26.4 per 100,000). In 2015, Grenada had the highest rates among men (104.1 per 100,000), whereas the lowest were found in Ecuador (30.4 per 100,000). The overall ASDR decreased from 80 to 44.2 per 100,000.

Throughout the LAC, men showed a statistically significant decrease in mortality (AAPC, –1.4%), with one joinpoint observed in 1994. Mortality rates between 1979 and 2015 decreased in 14 LAC countries. Chile, Colombia, and Uruguay had the largest decreases, whereas we observed a significant increase in St Vincent. The overall ASDR decreased from 80 to 44.2 per 100,000.

**Table 2:** Joinpoint analysis for stroke mortality trends in men in Latin America and the Caribbean, 1979–2015.


	TOTAL STUDY PERIOD		PERIOD 1		PERIOD 2		PERIOD 3	
			
	YEARS	APC (95% CI)	YEARS	APC (95% CI)	YEARS	APC (95% CI)	YEARS	APC (95% CI)

LAC	1979–2015	–1.4* (–1.7, –1.1)	1979–1994	–0.1 (–0.7, 0.5)	1994–2015	–2.3* (–2.6, –1.9)		

Antigua and Barbuda	1985–2015	–2.4 (–8.9, 4.6)	1985–1988	2.4 (–2.2, 7.3)	1998–2001	–29.3 (–64.6, 41.0)	2001–2015	0.0 (–3.2, 3.4)

Argentina	1979–2015	–2.1* (–2.6, –1.7)	1979–1992	–0.4 (–0.9, 0.2)	1992–2012	–4.0* (–4.3, –3.7)	2012–2015	2.9 (–2.3, 8.3)

Bahamas	1980–2013	–2.2* (–3.4, –0.9)						

Barbados	1979–2013	–2.1 (–5.6, 1.4)	1979–1995	–1.9* (–3.5, –0.3)	1995–2004	–9.2 (–20.7, 4.0)	2004–2013	5.1* (1.0, 9.2)

Belize	1980–2015	0.4 (–0.4, 1.3)						

Brazil	1979–2015	–1.5* (–2.2, –0.9)	1979–1988	–0.5 (–1.2, 0.3)	1988–1991	8.3 (–0.0, 17.4)	1991–2015	8.3 (–0.0, 17.4)

Chile	1980–2015	–2.4* (–2.5, –2.3)						

Colombia	1984–2015	–3.3* (–4.3, –2.3)	1984–1988	–15.5* (–20.5, –10.2)	1988–1995	3.5* (0.2, 6.9)	1995–2015	–3.0* (–3.5, –2.5)

Costa Rica	1980–2014	–1.8* (–3.1, –0.5)	1980–1988	4.4* (0.8, 8.2)	1988–2007	–5.4* (–6.4, –4.4)	2007–2014	1.3 (–3.0, 5.8)

Cuba	1979–2015	–0.8* (–1.1, –0.5)	1979–1986	0.4 (–1.0, 1.7)	1986–2015	–1.1* (–1.3, –0.9)		

Ecuador	1979–2015	–1.6 (–3.5, 0.3)	1979–1992	0.2 (–1.9, 2.4)	1992–1997	–8.2 (–19.2, 4.4)	1997–2015	–1.1 (–2.4, 0.2)

El Salvador	1981–2014	–1.8* (–3.0, –0.5)	1981–1992	4.2* (1.9, 6.5)	1992–2008	–6.8* (–8.1, –5.5)	2008–2014	1.4 (–3.6, 6.7)

Grenada	1985–2015	0.3 (–0.9, 1.4)						

Guatemala	1979–2015	0.8 (–1.7, 3.2)	1979–1989	–3.9 (–8.1, 0.5)	1989–1995	14.5* (1.2, 29.6)	1995–2015	–0.7 (–2.2, 0.8)

Guyana	1979–2013	–1.7* (–2.3, –1.1)						

Jamaica	1980–2011	–0.9* (–1.5, –0.3)						

Mexico	1979–2015	–0.5 (–1.1, 0.0)	1979–1989	3.5* (1.7, 5.3)	1989–2015	–2.0* (–2.4, –1.6)		

Nicaragua	2010–2015	–4.2* (–7.1, –1.2)						

Panama	1979–2015	–1.2 (–2.6, 0.3)	1979–2005	–0.6* (–1.1, –0.1)	2005–2010	–4.9 (–13.0, 4.1)	2010–2015	–0.4 (–6.5, 6.2)

Paraguay	1979–2014	–0.8 (–3.1, 1.6)	1979–1995	1.5* (0.3, 2.7)	1995–1998	–10.4 (–31.9, 17.8)	1998–2014	–1.1* (–2.2, –0.1)

Saint Lucia	1979–2014	–1.9* (–2.6, –1.2)						

St. Vincent	1982–2015	1.4* (0.5, 2.2)						

Suriname	1979–2014	–0.5 (–3.0, 2.1)	1979–1995	–3.0 (–5.9, 0.1)	1995–2004	11.8* (4.2, 20.0)	2004–2014	–6.6* (–11.2, –1.8)

Trinidad and Tobago	1979–2011	–1.5* (–2.9, –0.2)	1979–1982	8.6 (–4.2, 23.1)	1982–2003	–1.8* (–2.5, –1.1)	2003–2011	–4.4* (–7.0, –1.8)

Uruguay	1980–2015	–2.8* (–3.1, –2.4)	1980–2004	–1.6* (–1.9, –1.3)	2004–2015	–5.3* (–6.2, –4.4)		

Venezuela	1979–2013	–0.9 (–1.8, 0.0)	1979–1986	–2.3* (–4.4, –0.1)	1986–1992	1.8 (–2.8, 6.7)	1992–2013	–1.2* (–1.6, –0.8)


APC, annual percent change; CI, confidence interval. * P < 0.05 for change in trend.

For women, the highest rates in 1979 were recorded in Guyana (140.4 per 100,000), and the lowest in Guatemala (22.7 per 100,000). In 2015, we observed the highest female rate in St. Vincent (61 per 100,000) and the lowest in Ecuador (26.4 per 100,000) (***Table 3***). The overall ASDR decreased from 72.3 to 32.1 per 100,000.

**Table 3:** Joinpoint analysis for stroke mortality trends in women in Latin America and the Caribbean, 1979–2015.


	TOTAL STUDY PERIOD		PERIOD 1		PERIOD 2		PERIOD 3	
			
	YEARS	APC (95% CI)	YEARS	APC (95% CI)	YEARS	APC (95% CI)	YEARS	APC (95% CI)

LAC	1979–2015	–2.4* (–2.8, –2.0)	1979–1993	–1.1* (–1.7, –0.6)	1993–2010	–2.4* (–2.8, –2.0)	2010–2015	–5.9* (–8.2, –3.4)

Antigua and Barbuda	1985–2015	–0.6 (–5.1, 4.2)	1985–1992	6.9 (–3.7, 18.7)	1992–2012	–7.0* (–9.7, –4.3)	2012–2015	31.4 (–11.1, 94.3)

Argentina	1979–2015	–2.7* (–3.3, –2.2)	1979–1988	–0.4 (–1.3, 0.4)	1988–2013	–4.2* (–4.3, –4.0)	2013–2015	5.2 (–4.2, 15.6)

Bahamas	1980–2013	–2.4* (–3.6, –1.3)						

Barbados	1979–2013	–2.6* (–4.1, –1.2)	1979–1994	–2.4* (–3.7, –1.1)	1994–2001	–8.3* (–14.1, –2.3)	2001–2013	0.5 (–1.4, 2.5)

Belize	1980–2015	0.7 (–5.1, 6.8)	1980–2000	0.5 (–1.5, 2.6)	2000–2003	–13.2 (–56.6, 73.5)	2003–2015	4.7* (0.5, 9.1)

Brazil	1979–2015	–2.3* (–3.5, –1.2)	1979–1989	–3.0* (–4.0, –1.9)	1989–1992	6.2 (–7.6, 22.1)	1992–2015	–3.1* (–3.4, –2.8)

Chile	1980–2015	–2.9* (–3.8, –2.0)	1980–1992	–2.7* (–3.3, –2.1)	1992–1995	–6.5 (–16.0, 4.0)	1995–2015	–2.5* (–2.8, –2.2)

Colombia	1984–2015	–3.5* (–4.8, –2.3)	1984–1988	–17.4* (–22.6, –11.8)	1988–1993	6.6 (–0.1, 13.8)	1993–2015	–3.0* (–3.5, –2.5)

Costa Rica	1980–2014	–2.7* (–3.7, –1.7)	1980–1992	–1.2 (–2.8, 0.3)	1992–2007	–5.6* (–6.8, –4.4)	2007–2014	1.1 (–2.4, 4.8)

Cuba	1979–2015	–1.6* (–1.9, –1.3)	1979–1995	–0.8* (–1.2, –0.3)	1995–2015	–2.3* (–2.6, –1.9)		

Ecuador	1979–2015	–1.6 (–5.1, 2.0)	1979–1993	0.4 (–1.6, 2.4)	1993–1996	–13.1 (–43.7, 34.2)	1996–2015	–1.2 (–2.4, 0.1)

El Salvador	1981–2014	–2.3 (–5.0, 0.5)	1981–1990	4.5 (–6.2, 16.4)	1990–2014	–4.7* (–5.4, –4.0)		

Grenada	1985–2015	–1.1 (–2.4, 0.3)						

Guatemala	1979–2015	0.6 (–2.2, 3.4)	1979–1988	–5.1 (–11.0, 1.2)	1988–1995	13.6* (1.4, 27.4)	1995–2015	–1.1 (–2.9, 0.8)

Guyana	1979–2013	–0.7 (–1.5, 0.1)						

Jamaica	1980–2011	–1.1 (–2.8, 0.7)	1980–1983	8.0 (–10.2, 29.9)	1983–2011	–2.0* (–3.1, –0.9)		

Mexico	1979–2015	–0.9* (–1.6, –0.2)	1979–1988	3.6* (0.9, 6.3)	1988–2015	–2.3* (–2.8, –1.8)		

Nicaragua	2010–2015	–3.1 (–6.2, 0.0)						

Panama	1979–2015	–2.2 (–4.8, 0.4)	1979–1989	–2.4 (–5.1, 0.3)	1989–2001	0.2 (–7.4, 8.4)	2001–2015	–4.1* (–5.7, –2.5)

Paraguay	1979–2014	–0.7 (–2.7, 1.3)	1979–1995	1.4* (0.4, 2.5)	1995–1998	–9.8 (–28.7, 14.1)	1998–2014	–1.1* (–2.0, –0.2)

Saint Lucia	1979–2014	–1.8 (–3.6, 0.0)	1979–1987	4.2 (–3.6, 12.6)	1987–2014	–3.5* (–4.6, –2.5)		

St. Vincent	1982–2015	0.5 (–0.7, 1.7)						

Suriname	1979–2014	–0.8 (–2.6, 1.0)	1979–1995	–2.3* (–4.3, –0.1)	1995–2014	9.2* (3.9, 14.7)	2004–2014	–6.7* (–10.0, –3.4)

Trinidad and Tobago	1979–2011	–3.0* (–3.6, –2.5)	1979–1997	–1.7* (–2.4, –0.9)	1997–2011	–4.8* (–5.8, –3.8)		

Uruguay	1980–2015	–3.3* (–3.9, –2.7)	1980–1986	–1.2 (–3.7, 1.4)	1986–2005	–3.0* (–3.5, –2.5)	2005–2015	–5.2* (–6.3, –4.1)

Venezuela	1979–2013	–1.9* (–2.8, –1.1)	1979–1986	–3.6* (–5.6, –1.5)	1986–1992	1.4 (–3.2, 6.1)	1992–2013	–2.3* (–2.7, –1.9)


APC, annual percent change; CI, confidence interval. * P < 0.05 for change in trend.

Among LAC women, we recorded a steady and statistically significant decrease of –2.4% in the AAPC, with two joinpoints, 1993 and 2010. Mortality from stroke decreased in 12 LAC countries. The most pronounced decreases were observed in Colombia, Trinidad and Tobago and Uruguay. There was no significant increase for LAC women.

### Trends of mortality from stroke by region and income classification

In Caribbean countries the overall ASDR decreased from 113.6 to 59.2 per 100,000. We recorded a non-statistically significant decrease of –1.4% in the AAPC, with two joinpoint, 1998 and 2002. In Central America, the overall ASDR decreased from 34.4 to 32.3 per 100,000. We recorded a non-statistically significant decrease of –0.7% in the AAPC, with one joinpoint in 1981. In South America, the overall ASDR decreased from 75.7 to 34.8 per 100,000. We recorded a statistically significant decrease of –2.1% in the AAPC, with two joinpoints, 1982 and 2011 (***Table 4***).

**Table 4:** Jointpoint analysis for stroke mortality trends by Latin America and the Caribbean regions, 1979-2015.


	TOTAL STUDY PERIOD		PERIOD 1		PERIOD 2		PERIOD 3	
			
	YEARS	APC (95% CI)	YEARS	APC (95% CI)	YEARS	APC (95% CI)	YEARS	APC (95% CI)

Caribbean	1979–2015	–1.4 (–3.5, 0.8)	1979–1998	–0.3 (–1.4, 0.8)	1998–2002	–9.4 (–24.9, 9.2)	2002–2015	–0.4 (–2.4, 1.5)

Central	1979–2015	–0.7 (–2.4, 1.0)	1979–1981	22.3 (–10.8, 67.6)	1981–2015	–1.9* (–2.3, –1.5)		

South	1979–2015	–2.1* (–2.9, –1.4)	1979–1982	3.5 (–3.3, 10.8)	1982–2011	–1.9* (–2.1, –1.7)	2011–2015	–7.8* (–11.6, –3.7)

High income	1979–2015	–2.7* (–3.4, –2.0)	1979– 1992	–1.1* (–2.1, –0.1)	1992–2001	–5.0* (–7.1, –3.0)	2001–2015	–2.5* (–3.5, –1.6)

Middle income	1979–2015	–1.9* (–2.3, –1.5)	1979–2010	–1.1* (–1.2, –0.9)	2010–2015	–6.8* (–9.2, –4.2)		


APC, annual percent change; CI, confidence interval. * P < 0.05 for change in trend.

In high-income countries, the overall ASDR decreased from 110 to 36.9 per 100,000. We recorded a statistically significant decrease of –2.7% in the AAPC, with two joinpoints, 1992 and 2001. In middle-income countries, the overall ASDR decreased from 74.4 to 35.2 per 100,000. We recorded a non-statistically significant decrease of –1.9% in the AAPC, with one joinpoint in 2010 (***Table 4***).

## Discussion

This analysis of trends in mortality from stroke in LAC showed overall downward trends in both sexes between 1979 and 2015. According to the regional analysis, southern and high-income countries showed the steepest decreases. The largest decreases were found in Chile, Colombia, and Uruguay.

In general, mortality is linked to incidence and case-fatality. The decrease in stroke mortality has been attributed to reduced stroke incidence due to improvements in primary prevention and risk factor management, combined with enhancement in the acute management of stroke, leading to reduced case-fatality [14]. Likewise, the reduction in the incidence of intracerebral hemorrhage (ICH) in LAC in recent decades could have a great impact on reducing stroke mortality, considering the higher case fatality associated with this subtype of stroke (about 50%).

Two Brazilian studies have reported a significant reduction in the stroke incidence and mortality. In Matão, the age-adjusted incidence decreased by 39% (incidence rate ratio [IRR] 0.61; 95% CI 0.46–0.79) and mortality by 50% (IRR 0.50; 95% CI 0.31–0.94), whereas 30-day case-fatality did not significantly change between 2003–2004 and 2015–2016 (18.5 vs. 17.3%) [16]. Likewise, in Joinville, Brazil in the period 1995–2006, the incidence decreased by 27%, mortality decreased by 37% and the 30-day case-fatality decreased by 28% [5]. Conversely, stroke incidence in Chile increased from 140.1 to 163.4 per 100,000 inhabitants between 2005 and 2021, although the 30-day case-fatality decreased from 24.6% to 23.3% for the same period [6,17]. Based in these data, we can assume that the decrease in stroke mortality in LAC is due to a significant reduction in incidence and case-fatality of stroke in the last decade. In addition, we note the reduction of the incidence of ICH in the region. In particular, in Chile the incidence of ICH decreased from 27.6 per 100,000 inhabitants in 2005 to 17.9 per 100,000 inhabitants in 2021 [6,17].

Although most studies have demonstrated a decrease in early case-fatality after stroke, variations in the level of acute care may account for differences in the magnitude of improvement [18]. In this sense, the countries in LAC took longer than high-income countries to develop acute stroke care [11]. Likewise, the most cost-effective intervention to reduce mortality and disability is the admission of stroke patients to stroke units (SU) [19]; however, there are significant differences between LAC countries in the number of SU [11]. Although SU are available in all LAC countries, their number varied substantially from only one unit in Ecuador and Guatemala to 156 units in Brazil [11]. On the other hand, the access to reperfusion therapies, intravenous thrombolysis (IVT) and endovascular treatment (EVT) in LAC was very limited [11]. IVT for patients with acute ischemic stroke was available in all LAC countries, but only for a relatively small proportion of patients (usually <1%). An even smaller proportion of eligible patients received EVT [11]. In comparison, 7.3% of all stroke patients received IVT and 1.9% EVT in Europe [20]. A recent study reported that the number of stroke centers increased from 322 to 448 between 2000 and 2018 in Latin America, with a significant increase in the number of SU [8].

Our results also showed a higher reduction of stroke mortality in high-income LAC countries than in middle-income countries. In this sense, the Global Burden of Disease (GBD) study 2019 found that the age-standardized stroke-related mortality rate was 3.6 times higher in the World Bank low-income than in the high-income group [21]. Likewise, the PURE study reported that the rates of major cardiovascular events (including death from stroke) were lower in high-income countries than in middle-income countries (3.99 vs. 5.38 events per 1,000 person-years) in spite of a higher burden of cardiovascular risk factors. Case-fatality rates were also lowest in high-income countries in comparison with middle-income countries (6.5% vs. 15.9%) [22]. High-income countries have a greater use of preventive drugs and reperfusion therapies, better control of hypertension, and lower current smoking rates (all markers of better health care systems), which may mitigate this higher risk factor burden [22].

A reduction in the stroke mortality rate has also been reported in high-income countries. In the United States, for the period 2005–2015, the age-adjusted stroke death rate decreased 21.7% (from 48.0 to 37.6 per 100,000). The decline in men and women was similar (–21.9% and –21.5%, respectively) [23]. Additionally, in a recent study we reported a significant decrease in stroke mortality in the European Union, with an AAPC –4.2% in the period 1996–2015 [15].

Our general results are less pronounced than those reported by Shah et al., who analyzed trends in stroke mortality in Europe between 1980 and 2016. They found an AAPC of –2.7% for men and –2.7% for women, and a recent plateauing associated with hemorrhagic stroke and increases in ischemic stroke mortality in some countries [14]. However, the AAPC of –2.7% detected in high-income LAC countries is comparable to the reduction in stroke mortality rate reported in Europe and other high-income countries.

Our results also coincide with a systematic analysis of the GBD 2016 study that found a percentage change in age-standardized stroke mortality rates for the period 1990–2016 of –53.2% in Southern Latin America, –42.6% in Central Latin America, –54.9 in Andean Latin America, –28.3% in the Caribbean, and –55.5% in Tropical Latin America [1]. This study also found a reduction of –51.9% in high socio-demographic index (SDI) countries, and –38.2% in middle SDI countries [1]. These results are consistent with those of our study in relation to the higher decrease of stroke mortality in high-income LAC countries compared to middle-income countries. In this sense, it is remarkable that the joinpoint in 2012, in Chile and Uruguay, coincides with the year when these two countries entered the group of high-income countries according to the World Bank classification. Therefore, our results confirm the influence of country income level on stroke mortality. High-income countries have lower stroke mortality rates than middle-income and low-income countries despite a higher cerebrovascular risk factor burden.

This study has several strengths. To our knowledge, it is the most recent study analyzing stroke mortality trends in LAC. On the other hand, mortality data were extracted directly from an official database with no need to calculate mortality from death count and population data. Likewise, the Joinpoint regression software has been widely used to analyze mortality trends in cardiovascular and cerebrovascular disease [14,15,24,25]. In addition, this analysis method achieves a better fit compared to linear models, which reduce the trend to a single regression [26]. Our work also has limitations. The main limitation is the lack of data from some countries, where data were not provided for some years. For this reason, we decided not to extrapolate or impute, but to analyze only official data. Second, our study does not include within-country mortality rates given that regional variations in stroke mortality have been reported in some LAC countries [27]. Another limitation is that the data are not stratified according to major stroke pathological subtype (ischemic and hemorrhagic). Finally, we recognize that mortality trend studies only describe trends and do not seek to explain them [14]. This analysis needs further work to investigate the association between trends in stroke mortality and sociodemographic characteristics, such as measures of income and health care expenditure [14].

According to the United Nations, 7.8% of the LAC population was 65+ years old in 2015. For 2050 it is expected that this subgroup of people, who have the highest stroke risk, will increase to 19% [28]. Therefore, despite the decline in stroke mortality rates in LAC, the absolute number of deaths from stroke will not substantially decline and may even increase in the shorter and longer term due to the aging of national populations.

The GBD 2019 study found that the five leading risk factors for stroke are high blood pressure, high body mass index, high fasting plasma glucose, ambient particulate matter pollution and smoking [21]. On the other hand, the contribution of high systolic blood pressure, high body mass index, alcohol use, and air pollution to disability adjusted life years (DALYs) in Latin American countries were greater than those in high-income countries [11]. This could be one of the reasons for the lower decrease in stroke mortality (–1.9% AAPC) in LAC countries compared to high-income countries.

The INTERSTROKE Study found that ten potentially risk factors were collectively associated with about 90% of the population attributable risk (PAR) of stroke worldwide (93.2% of PAR in South America). However, there are important regional variations in the relative importance of most individual risk factors for stroke, which could contribute to worldwide differences in frequency and case-mix of stroke [9]. In the case of South America, hypertension, regular physical activity, and diet respectively account for 46.3%, 40.0% and 37.3% of the PAR. Therefore, global and region-specific programs are required to prevent stroke [9].

The Gramado Declaration signed in 2018 set priorities for stroke prevention, treatment, and research in Latin America countries [11]. Until 2020, only three Latin American countries had established a national plan for stroke: Brazil, Chile and Costa Rica. Uruguay, Paraguay, Colombia and Mexico are working with their governments to establish a program for stroke [8]. On the other hand, the HEARTS in the Americas, a PAHO program to reduce cardiovascular events through the control of hypertension, diabetes, dyslipidemia, and lifestyle modifications, is being implemented in several LAC countries [29].

## Conclusions

Stroke mortality has decreased in LAC, in both sexes, especially in southern and high-income countries. Our results could serve as a reference for the development of primary prevention and acute management of stroke policies focused on countries with higher mortality.

## Data Accessibility Statements

The data that support the findings of this study are openly available from the World Health Organization Mortality Database at *https://apps.who.int/healthinfo/statistics/mortality/whodpms/*, reference number [12].
